# Training on Addressing Patients’ Values (including Spirituality and Worldview) in Decision Making

**DOI:** 10.1192/j.eurpsy.2024.1654

**Published:** 2024-08-27

**Authors:** A. L. H. Peh, D. C. L. Teo, E. K. Ong, M. S. Q. Tan

**Affiliations:** ^1^Psychological Medicine, Changi General Hospital; ^2^Palliative Care, National Cancer Centre Singapore, Singapore, Singapore

## Abstract

**Introduction:**

Patients’ values are relevant in patient-centred care (PCC) as awareness and recognition of these can lead to better decision making and improved outcomes. Training in decision making is sorely lacking, especially in the area of spirituality and worldview.

**Objectives:**

Our poster describes a training workshop to provide such medical education to healthcare professionals. The half-day training covers: importance of addressing patients’ values in decision making; using decisional aids; role of spirituality and worldview of the patient.

**Methods:**

Clinicians of the hospital, including doctors, nurses and allied healthcare professionals were invited to attend the training. The evaluations by the participants for the workshops conducted in 2021-2023 were collated and presented.

**Results:**

Four workshops in 2021 to 2023 were conducted, with a total of 43 participants. We achieved overall ratings of above average and excellent in more than 80% of responses; content relevance and usefulness to work, presentation and facilitation were similarly rated. Most participants would recommend it to colleagues.

**Conclusions:**

The “Addressing Patients’ Values in Decision Making” workshop for clinicians will allow the hospital to promulgate a culture of quality care through patient engagement.

**Disclosure of Interest:**

None Declared

